# Longitudinal Analysis of Stroke Patients' Brain Rhythms during an Intervention with a Brain-Computer Interface

**DOI:** 10.1155/2019/7084618

**Published:** 2019-04-14

**Authors:** Ruben I. Carino-Escobar, Paul Carrillo-Mora, Raquel Valdés-Cristerna, Marlene A. Rodriguez-Barragan, Claudia Hernandez-Arenas, Jimena Quinzaños-Fresnedo, Marlene A. Galicia-Alvarado, Jessica Cantillo-Negrete

**Affiliations:** ^1^Electrical Engineering Department, Universidad Autónoma Metropolitana Unidad Iztapalapa, Mexico City 09340, Mexico; ^2^Division of Research in Medical Engineering, Instituto Nacional de Rehabilitación “Luis Guillermo Ibarra Ibarra”, Mexico City 14389, Mexico; ^3^Neuroscience Division, Instituto Nacional de Rehabilitación “Luis Guillermo Ibarra Ibarra”, Mexico City 14389, Mexico; ^4^Division of Neurological Rehabilitation, “Instituto Nacional de Rehabilitación Luis Guillermo Ibarra Ibarra”, Mexico City 14389, Mexico; ^5^Department of Electrodiagnostic, . National Institute of Rehabilitation, “Luis Guillermo Ibarra Ibarra”, Mexico City 14389, Mexico

## Abstract

Stroke is a leading cause of motor disability worldwide. Upper limb rehabilitation is particularly challenging since approximately 35% of patients recover significant hand function after 6 months of the stroke's onset. Therefore, new therapies, especially those based on brain-computer interfaces (BCI) and robotic assistive devices, are currently under research. Electroencephalography (EEG) acquired brain rhythms in alpha and beta bands, during motor tasks, such as motor imagery/intention (MI), could provide insight of motor-related neural plasticity occurring during a BCI intervention. Hence, a longitudinal analysis of subacute stroke patients' brain rhythms during a BCI coupled to robotic device intervention was performed in this study. Data of 9 stroke patients were acquired across 12 sessions of the BCI intervention. Alpha and beta event-related desynchronization/synchronization (ERD/ERS) trends across sessions and their association with time since stroke onset and clinical upper extremity recovery were analyzed, using correlation and linear stepwise regression, respectively. More EEG channels presented significant ERD/ERS trends across sessions related with time since stroke onset, in beta, compared to alpha. Linear models implied a moderate relationship between alpha rhythms in frontal, temporal, and parietal areas with upper limb motor recovery and suggested a strong association between beta activity in frontal, central, and parietal regions with upper limb motor recovery. Higher association of beta with both time since stroke onset and upper limb motor recovery could be explained by beta relation with closed-loop communication between the sensorimotor cortex and the paralyzed upper limb, and alpha being probably more associated with motor learning mechanisms. The association between upper limb motor recovery and beta activations reinforces the hypothesis that broader regions of the cortex activate during movement tasks as a compensatory mechanism in stroke patients with severe motor impairment. Therefore, EEG across BCI interventions could provide valuable information for prognosis and BCI cortical activity targets.

## 1. Introduction

Stroke is one of the leading causes of disability worldwide [1]. Ischemic stroke is the most common type and has a global incidence of approximately 11.6 million new cases per year [1]. One of the most disabling motor impairments produced by stroke is hemiparesis which is comprised by the complete or partial paralysis of one of the body sides, including the arm, leg, foot, and hand. Furthermore, after six months of the stroke's onset, only 35% of patients recover enough hand motor function to be able to use it in daily activities [2]. Therefore, research involving new therapies focused on stroke patients' upper limb rehabilitation is needed to increase the number of patients that achieve hand function recovery. Particularly, therapies based on robot assistive devices have shown potential for increasing stroke patients' neuroplasticity, the main recovery mechanism of stroke [2]. Some of these devices are specifically designed for upper limb motor rehabilitation by applying passive movement to stroke patients' paralyzed hand [3–6]. Another promising technology for upper limb rehabilitation of stroke patients is brain-computer interfaces (BCI). BCI allow control of external devices by decoding users' intentions from central nervous system sources such as the electroencephalogram (EEG) [7]. BCI systems are comprised by EEG signal acquisition, signal preprocessing, feature extraction, feature selection, classification, and external device communication stages [7]. Several paradigms allow BCI users to achieve control of the system, one of them is motor imagery/intention (MI), which is the mental rehearsal (motor imagery) or intention (motor intention) of movement execution and elicits similar cortical activations as actual movement [8]. MI elicits a power decrease and/or increase in alpha (8 to 13 Hz) and/or beta EEG frequencies (14 to 30 Hz) with respect to a baseline, known as event-related desynchronization or synchronization (ERD/ERS) [9]. Studies have described that stroke patients can still elicit ERD/ERS during MI of their paralyzed hand [10, 11] and during passive movement provided by robotic assistive devices [12]. Since ERD/ERS is associated with increased or decreased brain activity, it has been hypothesized that BCI systems controlled by hand MI and coupled to robotic assistive devices could be used to promote stroke patients' neuroplasticity processes, increasing the probability of upper limb function recovery [13, 14].

Although some studies have evaluated the effectiveness of a BCI coupled to robotic assistive devices for upper limb rehabilitation of stroke patients [15–18], to the authors' knowledge, none has evaluated the longitudinal changes and the relationship between upper limb motor recovery with brain rhythms recorded during a complete BCI intervention, in subacute stroke patients. Stroke patients' EEG, recorded during a BCI intervention, offers the possibly of evaluating this relationship, since changes in EEG brain rhythms have been related to neuroplasticity induced by different types of noninvasive stimulation. For example, Pellegrino et al. reported changes in EEG connectivity in stroke patients before and after a robotic hand therapy. The intervention lasted for 12 weeks, and changes in functional connectivity were reported to correlate with improvement in upper limb motor control [19]. In addition, Shindo et al. reported alpha and beta power differences in EEG electrodes placed above the somatosensory cortex of 8 stroke patients before and after 4 to 7 months intervention with a BCI coupled to a robotic hand orthosis. Half of the patients that showed more pronounced ERD over the affected hemisphere also had increased cortical excitability, measured by means of transcranial magnetic stimulation (TMS), implying a relationship between EEG power and brain plasticity [20]. Both studies reported preintervention and postintervention EEG changes; however, a trend analysis performed from several intraintervention EEG measurements could provide additional insights of neuroplasticity mechanisms involved in upper limb motor recovery. This trend analysis could comprise a longitudinal study of ERD/ERS features which could offer additional information of the neuroplasticity meaning of brain rhythms during a noninvasive intervention for stroke patients' hand rehabilitation.

The purposes of the present study are to describe changes in cortical activations across a BCI intervention and to analyze possible relationships between ERD/ERS trends and upper limb motor recovery in stroke patients. The BCI intervention was comprised by passive hand movement provided by means of a robotic hand orthosis driven by MI of the paralyzed hand of patients, undergoing an intervention as part of a larger study. EEG alpha and beta brain rhythms were recorded across 12 intervention sessions, and its association with upper limb motor recovery was analyzed.

## 2. Materials and Methods

### 2.1. Stroke Patients

Data of 9 stroke patients were included for the present study. Patients were recruited as part of a BCI validation study being conducted in the National Institute of Rehabilitation “Luis Guillermo Ibarra Ibarra” with the approval of its research committee. All patients read and signed an informed consent approved by the institute's ethical committee. Patients had an ischemic stroke diagnosis confirmed through neuroimaging by a neurologist. During the BCI intervention, patients were in the subacute phase of their stroke; therefore, no more than 10 months and no less than 2 months had passed since the stroke onset [21]. Patients were right handed before the stroke and had no previous history of neurological lesions and showed a cognitive performance with slight alterations in attention and memory processes, as well as adequate understanding of instructions, according to the neuropsychological test NEUROPSI [22].  Table 1 shows patients' information.

### 2.2. BCI System

**Figure 1:**
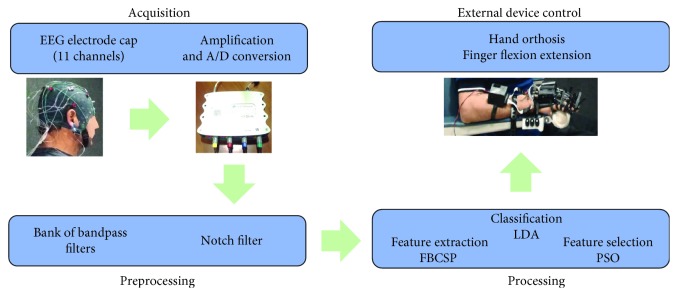
Stages of the BCI system employed for stroke patients' intervention.

The BCI system acquisition stage was comprised by a g.USBamp biosignal amplifier from g.tec and an electrode cap with 11 g.LADYbird electrodes placed in the *F*3, *F*4, *F*z, *P*3, *P*4, *P*z, *C*3, *C*4, *C*z, *T*3, and *T*4 positions of the international 10-20 system; ground electrode was placed in the *AF*z position and reference electrode in the right earlobe. All EEG recordings used for the BCI system were performed with electrode impedances below 5 K*Ω*. A computer monitor was also part of the system and allowed showing and playing visual and auditory cues to patients. Preprocessing and processing stages of the BCI system were programmed in a PC. The preprocessing stage of the BCI was comprised by a notch 60 Hz filter and bandpass filters in the following frequency bands: 8-12 Hz, 12-16 Hz, 16-20 Hz, 20-24 Hz, 24-28 Hz, and 28-32 Hz, all are of FIR type and order 30. The processing stage's feature extraction algorithm was a common spatial pattern (CSP) filter, applied to each one of the 6 frequency subbands [24], following a methodology similar to the Filter Bank Common Spatial Patterns (FBCSP) [25]. Features extracted with the spatial filters were selected using particle swarm optimization (PSO) and classified with linear discriminant analysis (LDA) [26]. The system's classification output was sent to a robotic hand orthosis through wireless communication. When activated by the BCI system, the robotic orthosis provided passive flexion followed by extension of the fingers of the hand. A more detailed description of the BCI system can be found in the work by Cantillo-Negrete et al. [27]. A depiction of the BCI system is shown in Figure 1.

### 2.3. BCI Intervention

**Figure 2:**
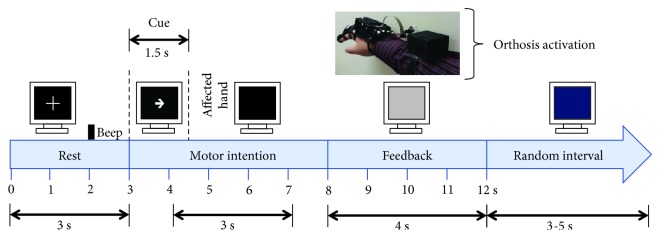
Structure of trials during the BCI intervention.

Patients underwent therapy with the BCI system during a 4-week intervention, with 3 sessions per week. Therefore, each patient had 12 BCI intervention sessions. Each session was comprised by 3 runs of 20 trials and lasted between 45 and 60 minutes. Patients rested for at least 3 minutes between runs. BCI sessions were conducted in a sound-attenuated room with the same illumination conditions and at the same time of the day. Patients were instructed to sit in a comfortable armed chair, with a computer monitor placed at approximately 1.5 m in front of them. The trials' time structure was based on the Graz paradigm [28] and was comprised by a first rest period of 3 s in which patients observed a white cross on the computer screen. At 2 s from initiating this rest period, a beep sound was played by the monitor's loudspeakers, notifying the patient that the task is about to begin. Three seconds after trials' onset, an arrow pointing to the direction of the patient's paralyzed hand appeared in the computer screen, signaling the patient to perform MI of the affected hand. This arrow lasted 1.5 s and afterwards disappeared with the monitor's screen turning black for another 3.5 s. During this time (arrow or black screen shown in the monitor), patients were instructed to perform MI of their paralyzed hand. After this time, the BCI system processed 3 s of EEG data segmented in 1-second length windows, from 4 to 7 seconds of the trial's structure. If 2 of these time windows were classified as MI, then the robotic orthosis was activated; if 1 or no time windows were classified as MI, then the robotic orthosis was not activated. Regardless of the orthosis activation, after 8 s from the trial onset, the screen turned grey for 4 s. Finally, each trial ended with a blue screen that lasted between 3 and 5 s, to prevent habituation, in which patients could blink their eyes, move, and rest.  Figure 2 shows a trial's structure.

### 2.4. Patients' Clinical Assessment

Clinical assessment of patients' upper extremity motor recovery after the BCI intervention was performed using the upper extremity Fugl-Meyer assessment (FMA-UE) [29], by applying the scale to each patient before and after the BCI intervention. FMA-UE ranks upper extremity motor recovery in a 0-66 scale, with a lower score representing a lower motor impairment. Differences in the scale's scores between preintervention and postintervention were used as clinical upper limb motor recovery markers.

### 2.5. EEG Signal Processing

Patients' raw EEG data recorded from each session were analyzed. Data were preprocessed with a 30^th^-order FIR filter from 8 to 32 Hz and a common average reference (CAR) spatial filter for reducing ground placement effects in EEG data [30]. For each trial and EEG channel, alpha and beta time-frequency features were computed by means of the Morlet wavelet transform as explained by Tallon-Baudry et al. [31]. In order to eliminate trials with excessive noise artifacts, the interquartile ranges from the 60 trials of each patients' session were computed. Trials that exceeded power values 3 times higher than the 3^rd^ quartile or 3 times lower than the 1^st^ quartile were regarded as outliers and eliminated from the session's trial sample. Less than 5% of the total recorded trials were eliminated with this procedure. Afterwards, percentage of ERD/ERS was computed for each trial by subtracting averaged power from the 3 s time interval that comprised the rest condition (0-3 s of the trials' time structure) from the trial's power during MI (4-7 s of the trials' time structure) and dividing it by the rest condition's averaged power and afterwards multiplying it by 100 [32]; this procedure is described in

(1) $$\begin{array}{c} \frac{\%\text{ERD}}{\text{ERS}}=\left(\frac{P_{\text{MI}}-P_{\text{rest}}}{P_{\text{rest}}}\right)*100, \end{array}$$

with *P*_MI_ being MI task's power and *P*_rest_ averaged power during the rest condition. Alpha features were extracted by averaging ERD/ERS values from the 8-13 Hz band and beta features by averaging values from 14-32 Hz. Afterwards, averaged MI-related ERD/ERS (4-7 s of the trials' time structure) were computed from alpha and beta bands. Therefore, for all patients' EEG channels to represent information from the same affected and unaffected hemisphere, regardless of the lesioned hemisphere, ERD/ERS for the left hemisphere's channels (*F*3, *C*3, *T*3, and *P*3) of patients with right hemisphere lesions were interchanged with those for the right hemisphere's channels (*F*4, *C*4, *T*4, and *P*4). This allowed the affected hemisphere's (AH) cortical activity to be shown over the left hemisphere's channels (*F*_AH_, *C*_AH_, *T*_AH_, and *P*_AH_) and unaffected hemisphere's (UH) activity to be shown over the right channels (*F*_UH_, *C*_UH_, *T*_UH_, and *P*_UH_). Grand average brain topographic maps of ERD/ERS were computed for each of the 12 intervention sessions, separately for alpha and beta frequency bands. To quantitatively analyze ERD/ERS across sessions, a trend analysis, proposed by López-Larraz et al., was performed by computing the slope of a least squares fitted linear regression model from the averaged ERD/ERS values of each session and sessions' time since stroke onset (stated in days), separately, for each channel and for alpha and beta bands [33].

### 2.6. ERD/ERS Association with Clinical Recovery

A stepwise linear regression model [34] was used to explore the relationship between clinical hand motor recovery and ERD/ERS trends across BCI intervention sessions. The predicted variable (dependent variable) was set as each patients' differences between preintervention and postintervention of the FMA-UE scores. Predictor variables (independent variables) were set as computed ERD/ERS slopes for each channel and patient. All possible combinations of initial predictor variables included in the model were assessed. Models were calculated separately for alpha and beta ERD/ERS slopes.

### 2.7. Statistical Analysis

ERD/ERS data were tested for Gaussian distribution by means of a Lilliefors-corrected Kolmogorov-Smirnov test (*α* = 0.05) [35]. After this test implied non-Gaussian distribution, statistically significant (*α* = 0.05) differences between ERD/ERS across sessions were assessed for each channel, separately for alpha and beta bands, with Friedman nonparametric tests for repeated measures design [36]. ERD/ERS linear trends across sessions were evaluated for significance by computing the Spearman correlation between mean ERD/ERS with time since stroke's onset, as performed by López-Larraz et al. [33]. Slope significance values were FDR corrected for multiple comparisons. For the stepwise linear regression analysis, only models that presented a statistically significant (*α* = 0.05) prediction of the dependent variable, measured by means of *p* values obtained from an *F* distribution and whose coefficients' confidence intervals (*α* = 0.05) did not included the 0 value, were included. If more than one model was statistically significant per frequency band, then the model with the lowest *p* value was presented for each band as advised by Draper and Smith [37]. All computations were performed using MATLAB® software from MathWorks.

## 3. Results

### 3.1. Patients' Clinical Assessment

Table 2 shows the FMA-UE scores obtained for each of the 9 patients that underwent the BCI intervention. Three patients (*P*3, *P*5, and *P*9) had score gains equal or higher than 3. Three patients (*P*4, *P*7, and *P*8) had score gains between 2 and 1, while 3 other patients (*P*1, *P*2, and *P*6) did not have score gains.

### 3.2. ERD/ERS Longitudinal Brain Maps

**Figure 3:**
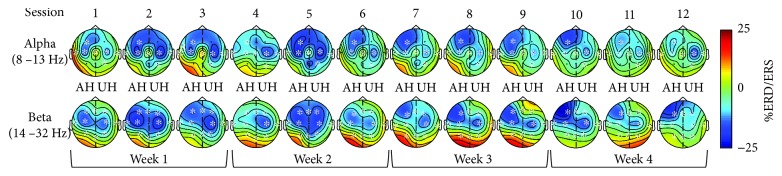
Grand average topographic maps of ERD/ERS during MI are observed across each session. Blue tones show ERD and red tones show ERS. All maps are plotted using the same scale. Affected (AH) and unaffected hemispheres (UH) are shown. Channels with significant differences across sessions are marked (^∗^).

Figure 3 shows grand average ERD/ERS topographic maps separately for alpha and beta bands. In the alpha band, significant (*p* < 0.05) differences across sessions were observed in AH frontal, central, and temporal electrodes (*F*_AH_, *C*_AH_, and *T*_AH_). In the UH, significant (*p* < 0.05) differences were observed in central, temporal, and parietal electrodes (*C*_UH_, *T*_UH_, and *P*_UH_). In the sagittal region, only the central channel (*C*_Z_) showed significant (*p* < 0.05) differences across sessions. In beta, all channels in the AH (*F*_AH_, *C*_AH_, *T*_AH_, and *P*_AH_) and UH (*F*_UH_, *C*_UH_, *T*_UH_, and *P*_UH_) presented significant (*p* < 0.05) differences across sessions, while only the central channel (*C*_Z_) of the sagittal region did not present significant (*p* < 0.05) differences. Therefore, for four channels in alpha and one in beta, significant ERD/ERS (*p* < 0.05) differences were not observed across sessions. Since maps showed ERD (cortical activations) mainly in frontal and central areas, Wilcoxon signed-rank tests were used to assess if significant (*α* = 0.05) differences were found between central and frontal ERD/ERS for each session. In both alpha and beta, the comparisons that showed consistent differences across sessions were the ones observed between *F*_AH_ and *C*_UH_. In alpha, *F*_AH_ and *C*_UH_ were not significantly different in sessions 1 to 6 and 12, while more pronounced (*p* < 0.05) ERD in *F*_AH_ compared to *C*_UH_ were observed in sessions 7 to 11, while in beta, *F*_AH_ and *C*_UH_ were not significantly different in sessions 1 to 6 and 9, while more pronounced (*p* < 0.05) ERD in *F*_AH_ compared to *C*_UH_ were observed in sessions 7, 8, 10, 11, and 12.

### 3.3. ERD/ERS Longitudinal Trends

**Figure 4:**
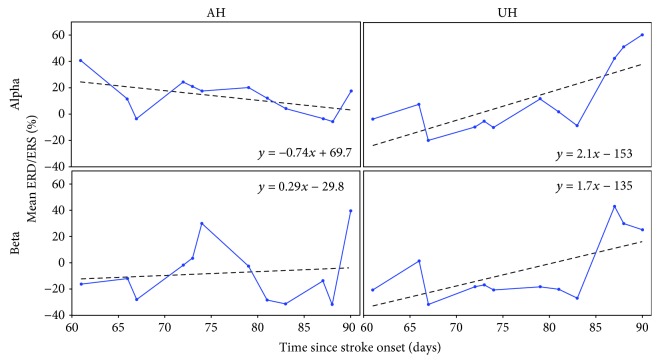
Example of linear trends, computed from average ERD/ERS across sessions and taking into account days since stroke onset. Slopes were calculated from central channels of the AH and UH of patient *P*5. Trends were computed separately for alpha and beta bands.

Central channel's (*C*_AH_ and *C*_UH_) linear trends computed from patient *P*5 mean %ERD/ERS across sessions can be observed in Figure 4. In alpha, a negative slope implied a trend towards ERD in the central AH, while a positive slope suggested a trend towards ERS in the central UH. In beta, trends towards ERS were observed in both AH and UH central channels.

Table 3 shows ERD/ERS slopes computed from each patient and channel calculated for alpha. A total of 45 slopes implied more pronounced ERD trends (negative slope), while the other 54 suggested less pronounced ERS (positive slope) across intervention sessions. Negative and positive ERD/ERS slopes that presented significant correlation with stroke's onset were observed in 24 and 36 channels, respectively. The trend with more pronounced ERD (lowest ERD/ERS slope) was observed in the UH parietal channel (*P*_UH_) of *P*5 (-2.81). The trend with less pronounced ERD (highest ERD/ERS slope) was observed in the UH central channel (*C*_UH_) of *P*5 (2.12).

Table 4 shows ERD/ERS slopes computed from each patient and channel calculated for beta. Approximately half of the slopes (49) implied more pronounced ERD trends (negative slope), while the others suggested (50) less pronounced ERS (positive slope) across intervention sessions. Negative and positive ERD/ERS slopes that presented significant correlation with stroke's onset were observed in 34 and 39 channels, respectively. The trend with more pronounced ERD (lowest ERD/ERS slope) was observed in the UH parietal channel (*F*_UH_) of *P*4 (-1.23). The trend with more pronounced ERD (highest ERD/ERS slope) was observed in the UH temporal channel (*T*_UH_) of *P*4 (2.09).

### 3.4. ERD/ERS Association with Clinical Recovery

Equations (2) and (3) show linear models computed with alpha and beta ERD/ERS trends' slopes, respectively. Negative coefficients in the models implied that a negative channel trend slope, observed when more pronounced ERD was presented across sessions, was associated with lower upper limb motor impairment after the BCI intervention. On the other hand, positive coefficients in the model suggested that a positive channel trend slope, which was observed when less pronounced ERD was presented across sessions, was associated with lower upper limb motor impairment after the BCI intervention. The intercept term shows how much changes in motor impairment could be observed if a patient did not present ERD/ERS trends in channels included in the model (zero-magnitude slopes).

Equation (2) shows the linear model, obtained through stepwise regression that best fitted stroke patient's hand motor recovery for the alpha band (*p* = 0.02). Channels from the frontal and temporal AH (*F*_AH_ and *T*_AH_), frontal sagittal (*F*z), and parietal UH (*P*_UH_) regions were included in the model. Coefficients' confidence intervals (*α* = 0.05) were not zero inclusive. The model's adjusted *R*^2^ was of 0.83, implying that the model successfully predicts 83% of FMA-UE scores' variability. The alpha model implies that trends across the intervention of more pronounced ERD in the *F*_AH_ and *P*_UH_, coupled to less pronounced ERD in the *T*_AH_ and *F*z, are associated with less upper limb motor impairment at the end of the BCI intervention. The intercept term of the model implies an average gain of 0.89 points in patients' post-BCI intervention FMA-UE, if no changes of included channels' ERD/ERS were presented across the intervention sessions (channels' slopes included in the model equal to zero).

(2) $$\begin{array}{c} {\text{FMUE}}_{\text{post}}-{\text{FMUE}}_{\text{pre}}=-\left(8.64\left[-13.8,-3.4\right]\right)\left(F_{\text{AH}}\right)+\left(3.9\left[1.1,6.8\right]\right)\left(T_{\text{AH}}\right)\ldots +\left(10.19\left[4.8,15.1\right]\right)\left(F_{\text{z}}\right)-\left(3.16\left[-4.7,-1.6\right]\right)\left(P_{\text{UH}}\right)+0.89. \end{array}$$

Equation 3 shows the linear model, obtained with stepwise regression, that best fitted stroke patients' hand motor recovery for the beta band (*p* = 0.001). The ERD/ERS slopes of the parietal (*P*_AH_) channel of the AH(PAH), coupled to the parietal sagittal (*P*z) and frontal (*F*_UH_) and central (*C*_UH_) UH channels were included in the model. All coefficient confidence intervals (*α* = 0.05) were not zero inclusive. The model's adjusted *R*^2^ was of 0.96, implying that the model successfully predicts approximately 96% of patients' FMA-UE scores' variability. The beta model implies that more pronounced ERD in *F*_UH_ and *P*z regions, coupled to less pronounced ERD in *P*_AH_ and *C*_UH_, are associated with less motor impairment at the end of the BCI intervention. The intercept term of the model was of 0.7; therefore, if no changes of included channels' mean ERD/ERS were presented across the intervention session (channels' slopes included in the model equal to zero), patients would have a 0.7 increase in their FMA-UE scores after the BCI intervention.

(3) $$\begin{array}{c} {\text{FMUE}}_{\text{post}}-{\text{FMUE}}_{\text{pre}}=\left(4.11\left[2.9,5.3\right]\right)\left(P_{\text{AH}}\right)-\left(6.25\left[-7.8,-4.8\right]\right)\left(P\text{z}\right)\ldots -\left(3.55\left[-4.4,-2.7\right]\right)\left(F_{\text{UH}}\right)+\left(1.28\left[0.8,1.8\right]\right)\left(C_{\text{UH}}\right)+0.7. \end{array}$$

## 4. Discussion

Cortical activation differences were observed in several regions across sessions of the BCI intervention. In both alpha and beta, activation changes were observed over the somatosensory cortex, which could be expected since MI-BCI systems coupled to robotic assistive devices have shown to elicit activity above this area in stroke patients [15, 27]. However, these central area significant changes across sessions were not associated in all patients with time since stroke onset. Other areas such as frontal, temporal, and parietal regions also showed significant changes across sessions in alpha and/or beta and in some patients these changes presented a significant association with time since stroke. This implied that regions usually not associated with motor tasks could be recruited in stroke patients during MI of their impaired upper limb. This is also reinforced by the observed evolution of compared activity in frontal AH and central UH across sessions, since similar activations were more likely to be observed over these regions in alpha and beta in earlier sessions and afterwards changed towards more pronounced activations in frontal AH compared to central UH, in later sessions of the intervention. This could suggest that the frontal region of the AH could have been recruited, during the contralateral MI task, as a compensatory mechanism enhanced across the intervention. Recruitment of parietal and frontal regions has also been reported during stroke patients' evolution using fMRI by Ward et al. and hypothesized a possible enlargement of the motor region for compensating damage of the corticospinal tract [38]. Fewer patients' cortical areas in the alpha band showed activation trends across sessions, which had a significant association with stroke's onset, compared to beta. This could imply that a BCI intervention in stroke patients is more likely to elicit beta band modulation across time. It has been theorized that beta oscillations are associated with neural networks that propagate activity between primary motor cortex and muscles [39], while alpha has been related to motor information processes, such as learning of motor tasks [40]. Therefore, less ERD/ERS significant trends associated with time since stroke onset in alpha could be related to these processes, since patients' changes in alpha activity across sessions could be more associated with learning of the MI task. On the other hand, beta could reflect changes in motor cortex information processing within the corticospinal tract, as a consequence of neuroplasticity mechanisms, and thus could have a closer relationship with stroke time evolution compared to alpha oscillations. A similar hypothesis was also suggested by Gandolfi et al. since alpha and beta changes were also presented across a BCI intervention in a single stroke patient. The authors proposed that the alpha band was related with MI training processes, which could have aided to modulate beta, associated with corticospinal excitability [41].

An association between alpha ERD/ERS trends in frontal, temporal, and parietal regions with upper limb motor recovery was observed. However, the model's significance is less than the one recommended for linear regression analysis [37] and its prediction of upper limb recovery is moderate; therefore, it needs to be further confirmed with a higher sample of patients. A possible reason for this moderate association could be that alpha rhythm evolution across sessions is more related to the learning of the MI task, rather than to upper limb motor recovery, as previously suggested [39, 40, 42]. Interestingly, the alpha model did not include activity over electrodes directly located above the somatosensory cortex; this could also highlight the need for a larger sample of patients, to further assess somatosensory cortex alpha oscillation's association with upper limb motor recovery. On the other hand, an association with a recommended significance [36], between beta cortical activity trends across sessions in frontal, central, and parietal areas, with upper limb motor recovery, was observed. The model included the central UH implying that less pronounced cortical activity across sessions in this region was related to upper limb motor recovery during the intervention. However, it did not include an association between motor impairment with central AH cortical activity across sessions. Kaiser et al. reported in the alpha band of 27 stroke patients (which performed MI of their affected hand) that less pronounced cortical activation (less pronounced ERD) in the central UH, during patients' affected hand MI, was related to less motor impairment, while no association was found between cortical activations in the central AH with motor impairment [11]. Although, Kaiser et al. only reported similar central region observations in alpha, both alpha and beta have been associated with motor-related processes. Therefore, both Kaiser et al. observations and those of the present study's suggest that less pronounced central UH cortical activity could be related to less motor impairment, while AH activity could not be as associated with motor impairment. A possible explanation for these similar observations could be that it is more likely that patients' UH somatosensory cortex becomes less involved in ipsilateral motor processes as the corticospinal tract becomes more functional, compared to the possibility of the AH somatosensory cortex becoming more involved in contralateral motor processes, due to lesion heterogeneity presented in stroke. This hypothesis is reinforced by the observations reported by Lotze et al. in well-recovered subcortical stroke patients using MRI-derived measurements and TMS, since higher AH corticospinal tract integrity was associated with less pronounced activity in areas within the UH motor cortex [42]. The present study's beta model also implies that activation trends in areas usually not related with motor processes such as frontal and parietal regions could be associated with upper limb motor recovery after stroke. The model suggests that higher cortical activity trends (more pronounced ERD) in the UH frontal and parietal sagittal regions, combined with lower activity trends (more pronounced ERS) in parietal AH and central UH, were associated with higher motor recovery during the intervention. This could imply that an enlargement of the motor area, by including frontal and parietal regions, during motor-related processes, could be a neuroplasticity mechanism for improving upper limb function. Therefore, different degrees of activation in areas less related with motor processing, implied by the beta model, further reinforce previously observed enlargement of motor-processing areas during stroke recovery [38]. In addition, involvement of larger AH and UH areas during motor tasks has been described as a possible compensatory mechanism in patients with severe stroke motor impairment as reviewed by Cassidy and Cramer [43]. This could possibly explain the association of these regions in the present study, with upper limb motor impairment, as implied by alpha and beta linear models, since most patients included in the present study had moderate to severe upper limb stroke impairment. This is reinforced by the observations reported by Rondina et al. when predicting motor recovery in patients with severe stroke upper limb impairment, using features extracted from structural MRI and machine learning, to classify good or poor motor recovery, and suggested that prediction accuracy increased when larger areas of the somatosensory cortex were included in the model [44]. Furthermore, the longitudinal observations of Park et al. in stroke patients' resting state functional connectivity, computed from fMRI, reported connectivity changes comprising frontal and parietal cortex AH regions [45], reinforcing the hypothesis that normally non-motor-related regions could play a significant role during stroke recovery.

It has also been reported that interhemispheric differences in beta, between homologous somatosensory cortex areas, could play an important role in stroke motor recovery processes. For example, Shiner et al. reported an association between higher central AH and lower UH cortical activity in stroke patients' beta band, with lower motor impairment using magnetoencephalography, a high spatial and temporal resolution acquisition modality [46]. Furthermore, Pichiorri et al. reported that interhemispheric connectivity measurements, computed from EEG over somatosensory cortex regions in lower beta, can be associated with corticospinal integrity, evaluated by means of TMS [47]. Although the present work did not explore connectivity measurements among interhemispheric homologous areas, the beta model in the present work included the somatosensory cortex in the UH and implied that less beta cortical activity in this region was associated with higher motor recovery. Therefore, a trend towards less activity in the UH somatosensory cortex could be related with the recovery of interhemispheric balance across the BCI intervention. This could further reinforce the hypothesis of an association between interhemispheric differences with upper limb motor recovery; however, a larger sample and functional connectivity measurements should be computed in order to further assess this relationship.

According to the FMA-UE, patients' motor recovery was heterogenous along the sample. After the BCI intervention, 6 of 9 patients showed gains in upper limb motor recovery, while 2 patients showed no gains and one patient showed a decrease. All FMA-UE gains were in the range (4.55 ± 6.07) reported by Ang et al. with a 1-month intervention with a BCI coupled to a robotic assistive device [16]. Two-thirds of the patients with higher FMA-UE gains were in the range of other BCI stroke intervention studies, such as the one reported by Frolov et al. (5.25 ± 4.5) with a 1.5-month intervention [17] and Ramos-Murguialday et al. (3.4 ± 2.2) with a 1-month intervention [18]. Therefore, patients' clinical assessments show that the BCI intervention allowed most patients to achieve some degree of upper extremity motor recovery and that these recoveries are similar to the ones reported with other BCI interventions.

The present study has limitations that need to be acknowledged. One of them is the limited spatial resolution of EEG. This limited resolution, although acceptable for BCI applications, makes the association of more specific brain regions with upper limb recovery unfeasible, requiring the use of modalities like fMRI for further description of the evolution of brain rhythms across BCI interventions. Another limitation is the small sample of 9 patients' data, with heterogenous motor impairment (9 to 59 in the FMA), analyzed in the current study. This limited sample makes necessary the confirmation in a higher sample of patients of the presented regression models' association with upper limb motor impairment, especially the model computed from alpha ERD/ERS trends which showed lower than recommended significance. Also, most patients in the present study presented moderate to severe upper limb motor impairment and computed linear models could be mostly applicable to patients with these degrees of impairment. Therefore, studies that include a more balanced sample of patients with different degrees of stroke upper limb impairment are still needed for a more complete description of associations between upper limb impairment and brain rhythms. Taking into account these limitations, the present study's beta rhythm-described associations with upper limb motor recovery, which is thought to be more involved in closed-loop motor training processes [39], could bring more insight to the neuroplasticity mechanisms associated to good or poor upper limb recovery prognosis. This is important for establishing EEG brain rhythm longitudinal analysis as a complementary tool to other clinical assessments, for stroke patients' upper limb function prognosis. Also, specifically targeting trends and regions of AH and UH cortical activations during hand rehabilitation interventions, such as the ones implemented with BCI systems coupled to robotic devices, could potentially increase the number of patients that can achieve good rehabilitation outcomes.

## 5. Conclusion

This study presents a trend analysis of stroke patients' cortical activity during a BCI intervention aimed for hand rehabilitation. EEG trends in alpha seemed to be moderately related with time since stroke onset and recovery of upper limb motor function, probably reflecting neuroplasticity effects related to learning of the hand motor tasks. On the other hand, EEG trends in beta showed a higher association with time since stroke onset, compared to alpha, and a strong association with upper limb motor recovery. These beta band changes in hemispheres' nonhomologous activity along the BCI intervention suggested that longitudinal measurements could be associated with motor recovery of the upper limb. Although these findings need to be further confirmed with studies with higher spatial resolution and larger patient samples, it can be inferred that longitudinal analysis of EEG brain rhythms during stroke patients' hand rehabilitation interventions could provide valuable clinical information for both stroke prognosis and BCI intervention goals.

## Figures and Tables

**Table 1:** Clinical and demographic information of stroke patients' data included in the present study. Each patient's time since the beginning of the BCI of intervention, relative to stroke onset, and time at the end of the BCI intervention is shown. Percentage of infarct in regions related to the middle cerebral artery was assessed using the ASPECTS scale [23].

Patients' identifier	Age (years)	Gender	BCI intervention period relative to stroke onset (days)	Paralyzed hand	Lesion, type, and location of the affected area	Percentage of infarct in the middle cerebral artery
*P*1	54	Female	280 - 302	Right	Subcortical. L. lentiform nucleus, L. internal capsule, and L. thalamus	50%
*P*2	85	Female	111 - 137	Left	Subcortical. R. pontine tegmentum	NM
*P*3	58	Female	190 - 222	Right	Subcortical. L. lentiform nucleus and L. internal capsule	30%
*P*4	54	Female	176 - 204	Left	Cortical-subcortical. R. insula, R. lentiform nucleus, and R. internal capsule	40%
*P*5	43	Male	61 - 90	Left	Subcortical. R. pontine tegmentum	NM
*P*6	48	Male	99 - 125	Right	Subcortical. L. internal capsule	20%
*P*7	53	Male	127 - 156	Right	Cortical. L. insula	20%
*P*8	63	Male	260 - 285	Right	Subcortical. L. lentiform nucleus and L. internal capsule	20%
*P*9	65	Male	119 - 142	Left	Subcortical. R. internal capsule and R. thalamus	10%
Mean (±STD)	59.9 (±2.8)		158 (±74) – 185 (±73)			

NM: not measured if location did not comprise the middle cerebral artery; L.: left; R.: right.

**Table 2:** FMA-UE scores for 9 patients. Score ranges from 0 to 66; higher score's values imply lesser upper limb motor impairment.

Patient	Pre-BCI intervention	Post-BCI intervention	Intervention difference
*P*1	12	12	0
*P*2	13	13	0
*P*3	9	12	3
*P*4	11	12	1
*P*5	32	36	4
*P*6	15	14	-1
*P*7	16	17	1
*P*8	59	61	2
*P*9	16	20	4

**Table 3:** Slopes of ERD/ERS computed for each patient in the alpha band and for each AH (*F*_AH_, *C*_AH_, *T*_AH_, and *P*_AH_), sagittal (*F*z, *C*z, and *P*z), and UH (*F*_UH_, *C*_UH_, *T*_UH_, and *P*_UH_) channels. Slopes computed from ERD/ERS with a significant correlation with time since stroke onset (^∗^) are shown.

Patient	*F*_AH_	*C*_AH_	*T*_AH_	*P*_AH_	*F*z	*C*z	*P*z	*F*_UH_	*C*_UH_	*T*_UH_	*P*_UH_
*P*1	0.73^∗^	-0.05	0.17	0.52^∗^	0.41^∗^	0.10	0.13	0.65^∗^	0.43^∗^	0.43^∗^	-0.28
*P*2	0.09	0.34^∗^	0.00	0.13	0.18	0.03	0.07	-0.19	0.32^∗^	-0.20^∗^	0.22^∗^
*P*3	1.01^∗^	-0.14	0.34^∗^	0.60^∗^	0.98^∗^	0.20^∗^	0.51^∗^	0.69^∗^	0.22^∗^	0.25^∗^	0.22^∗^
*P*4	-0.29^∗^	-0.27^∗^	-0.13	-0.41^∗^	-0.19	-0.44^∗^	-0.30^∗^	-1.10^∗^	-0.40^∗^	-0.13^∗^	-0.02
*P*5	-0.38^∗^	-0.74^∗^	0.35^∗^	-1.05^∗^	-1.03^∗^	-2.03^∗^	-0.46	-0.92^∗^	2.12^∗^	-1.63^∗^	-2.81^∗^
*P*6	0.23^∗^	0.47^∗^	0.35^∗^	-1.12^∗^	-0.02	-0.80	-0.11	-0.22^∗^	1.14^∗^	-0.19	0.45^∗^
*P*7	0.09	0.66^∗^	-0.52^∗^	0.43^∗^	0.44^∗^	0.10	-0.31^∗^	0.17^∗^	0.50^∗^	0.68	0.62^∗^
*P*8	-0.16	-0.15	-0.07	-0.32^∗^	-0.11	-0.07	0.18	-0.11	-0.55^∗^	-0.03	-0.25^∗^
*P*9	0.49^∗^	0.06	0.71	0.65	0.54^∗^	0.74^∗^	-0.16	-0.09	0.06	0.93^∗^	0.32

**Table 4:** Slopes of ERD/ERS computed for each patient in the beta band and for each AH (*F*_AH_, *C*_AH_, *T*_AH_, and *P*_AH_), sagittal (*F*z, *C*z, and *P*z), and UH (*F*_UH_, *C*_UH_, *T*_UH_, and *P*_UH_) channels. Slopes computed from ERD/ERS with a significant correlation with time since stroke onset (^∗^) are shown.

Patient	*F*_AH_	*C*_AH_	*T*_AH_	*P*_AH_	*F*z	*C*z	*P*z	*F*_UH_	*C*_UH_	*T*_UH_	*P*_UH_
*P*1	-0.02	0.10^∗^	0.05	0.10^∗^	0.24^∗^	0.08	0.13^∗^	-0.02	0.04	0.25^∗^	0.10
*P*2	0.69^∗^	1.05^∗^	0.24^∗^	0.99^∗^	0.69^∗^	1.36^∗^	0.77^∗^	0.32^∗^	0.58^∗^	0.69^∗^	0.62^∗^
*P*3	-0.42^∗^	-0.58^∗^	-0.50^∗^	-0.05^∗^	-0.02	-0.08	-0.24^∗^	-0.28^∗^	-0.06	-0.36^∗^	-0.46^∗^
*P*4	1.37^∗^	-0.17	-0.03	1.03^∗^	2.02^∗^	0.42^∗^	1.24^∗^	-1.23^∗^	-0.35	-0.26	0.17
*P*5	0.24	0.29	0.50^∗^	-0.07	0.04	0.05	0.10^∗^	-0.54^∗^	1.68^∗^	0.05	-0.47^∗^
*P*6	-1.11^∗^	-0.90^∗^	-0.81^∗^	-1.09^∗^	-0.52^∗^	-0.97^∗^	-0.46^∗^	-0.12^∗^	-0.34^∗^	-0.31^∗^	-0.44^∗^
*P*7	0.41^∗^	0.93^∗^	-0.17^∗^	0.97^∗^	0.90^∗^	0.54^∗^	0.44^∗^	0.88^∗^	1.87^∗^	2.09^∗^	0.85^∗^
*P*8	-0.03	0.46^∗^	0.50^∗^	-0.23^∗^	-0.23^∗^	-0.34^∗^	-0.30^∗^	-0.21^∗^	-0.46^∗^	-1.13^∗^	-0.16^∗^
*P*9	-0.12	-0.22^∗^	0.75^∗^	0.28^∗^	-0.27	-0.58^∗^	-0.24^∗^	-0.23	-0.03	1.12^∗^	0.08

## Data Availability

The EEG data used to support the findings of this study are restricted by the National Institute of Rehabilitation Luis Guillermo Ibarra Ibarra ethics committee, in order to protect patient privacy. Data are available from Dr. Jessica Cantillo-Negrete for researchers who meet the criteria for access to confidential data.
